# High tibial osteotomy results in improved frontal plane knee moments, gait patterns and patient-reported outcomes

**DOI:** 10.1007/s00167-019-05644-7

**Published:** 2019-08-05

**Authors:** Gemma M. Whatling, Paul R. Biggs, David W. Elson, Andrew Metcalfe, Chris Wilson, Cathy Holt

**Affiliations:** 1 Cardiff School of Engineering, College of Physical Sciences and Engineering, Cardiff University, Queens Buildings, The Parade, Cardiff, C24 3AA UK; 2 Biomechanics and Bioengineering Research Centre Versus Arthritis, Cardiff University, Cardiff, UK; 3 Queen Elizabeth Hospital, Gateshead, UK; 4 University Hospital of Wales, Cardiff, UK; 5 Warwick Clinical Trials Unit, Warwick Medical School, University of Warwick, Coventry, UK

**Keywords:** Gait, Osteoarthritis, Knee, Loading, High tibial osteotomy

## Abstract

**Purpose:**

The purpose of this study was to quantify changes in knee loading in the three clinical planes, compensatory gait adaptations and patient-reported outcome measures (PROMS) resulting from opening wedge high tibial osteotomy (HTO).

**Methods:**

Gait analysis was performed on 18 participants (19 knees) with medial osteoarthritis (OA) and varus alignment pre- and post-HTO, along with 18 controls, to calculate temporal, kinematic and kinetic measures. Oxford Knee Score, Knee Outcome Survey and visual analogue pain scores were collected. Paired and independent sample tests identified changes following surgery and deviations from controls.

**Results:**

HTO restored frontal and transverse plane knee joint loading to that of the control group, while reductions remained in the sagittal plane. Elevated frontal plane trunk sway (*p* = 0.031) and reduced gait speed (*p* = 0.042), adopted as compensatory gait changes pre-HTO, were corrected by the surgery. PROMs significantly improved (*p* ≤ 0.002). Centre of pressure (COP) was lateralised relative to the knee post-HTO (*p* < 0.001). Energy absorbed in the sagittal plane significantly increased post-HTO (*p* = 0.007), whilst work done in the transverse plane reduced (*p* ≤ 0.008). Pre-operative gait deviations from the control group that were retained post-HTO included smaller sagittal (*p* = 0.003) knee range of motion during gait, greater stance duration (*p* = 0.008) and altered COP location (anterior to the knee) in early stance (*p* = 0.025).

**Conclusions:**

HTO surgery restored frontal and transverse plane knee loading to normal levels and improved PROMs. Gait adaptations known to reduce knee loading employed pre-HTO were not retained post-HTO. Some gait features were found to differ between post-HTO subjects and controls.

**Level of evidence:**

II

## Introduction

Medial knee osteoarthritis (OA) and associated varus alignment alters knee loading and gait patterns. It is of clinical importance to determine whether normal biomechanics are restored following corrective realignment surgery.

During the stance phase of gait, the ground reaction force (GRF) passes medially to the knee joint centre and the medial compartment bears the greatest proportion of load [2, 35]. This is exacerbated by varus knee deformity where patients are prone to develop more severe OA if the mechanics are not corrected [20, 34], evidenced by an increased risk of medial compartment joint space narrowing, osteophytes [36] and increased rate of medial tibial cartilage volume loss [37] in people with varus deformity.

High tibial osteotomy (HTO) surgery [22] corrects varus malalignment and this unloads the medial compartment by lateralising the weight bearing line.

Peak external knee adduction moment (EKAM) is a surrogate measure of medial knee loading [1, 32, 34, 39], as it correlates highly with internal medial contact forces in early stance [24, 40]. Opening wedge HTO reduces an initially elevated peak EKAM to a level lower than that observed in control subjects [6, 7, 14, 26, 27, 31, 33].

Angular impulses, the integrals of the external moment curves, also provide useful measures of loading, combining both magnitude and duration into one variable. Knee Adduction Angular Impulse (KAAI) correlates moderately with changes in medial-to-lateral load impulse ratio [5] and is associated with medial tibial cartilage volume loss [4] and OA grade [38].

Studies to date have mostly focused on changes in the peak EKAM and KAAI. However, this neglects important contributions to loading at the knee that can be measured within the other clinical planes, that can be further influenced by gait alterations adopted by the patient.

The primary aim of this study was to determine the key changes in knee kinetics, in three clinical planes, during the stance phase of gait in patients before and after HTO. It was hypothesised that pre-operatively, elevated frontal plane loading would be accompanied by altered loading in the sagittal and transverse planes relative to controls. Furthermore, it was hypothesised that HTO surgery would restore frontal plane loading to that of healthy controls whilst also affecting loading in the sagittal and transverse planes. The secondary aims were to identify gait adaptations employed prior to HTO that are known to affect loading at the knee, to report changes to these following HTO, along with changes in patient-reported outcomes. It was hypothesised that patient-reported function would improve, and that gait alterations observed pre-HTO would return to normal after surgery.

## Methods

Eighteen participants (19 knees) with medial compartment OA and varus alignment were recruited from the out-patient clinic of the senior surgeon on this paper (CW). Patients were included if they were between the ages of 18 and 80 and listed for medial opening wedge HTO. Patient did not pass initial screening if they were unable to provide informed consent, had neurological or visual conditions affecting movement or a previous injury to the joint under investigation that the treating clinician deemed unsuitable. The extent of OA was determined using the Kellgren–Lawrence (KL) [23] radiographic score and varus alignment calculated as the mechanical tibiofemoral angle (mTFA) from long leg weight bearing radiographs. Medial opening wedge HTO surgery was used to correct varus deformity using standard surgical approaches and planning [8]. In KL4 cases where bone-on-bone arthritis was identified, the intended correction was selected at 62.5% [15]. For lesser degrees of arthritis, the intended correction was neutral at 50%. The osteotomies were fixed with either Tomofix (*n* = 16) or Puddu (*n* = 1) plates, shown to have similar biomechanical properties [16], or an iBalance device (*n* = 2). Eighteen subjects with no lower limb pathology were also recruited from University staff, students and community using advertisements, forming a non-pathological (NP) control cohort. One leg was selected at random from NP participants to ensure 9 left and 9 right legs were used in the analyses. Approval for this work was granted by the Wales Research Ethics Committee 3 (10/MRE09/28) and Cardiff and Vale University Health Board. Written informed consent was obtained from each participant prior to data collection.

### Gait analysis

**Fig. 1:**
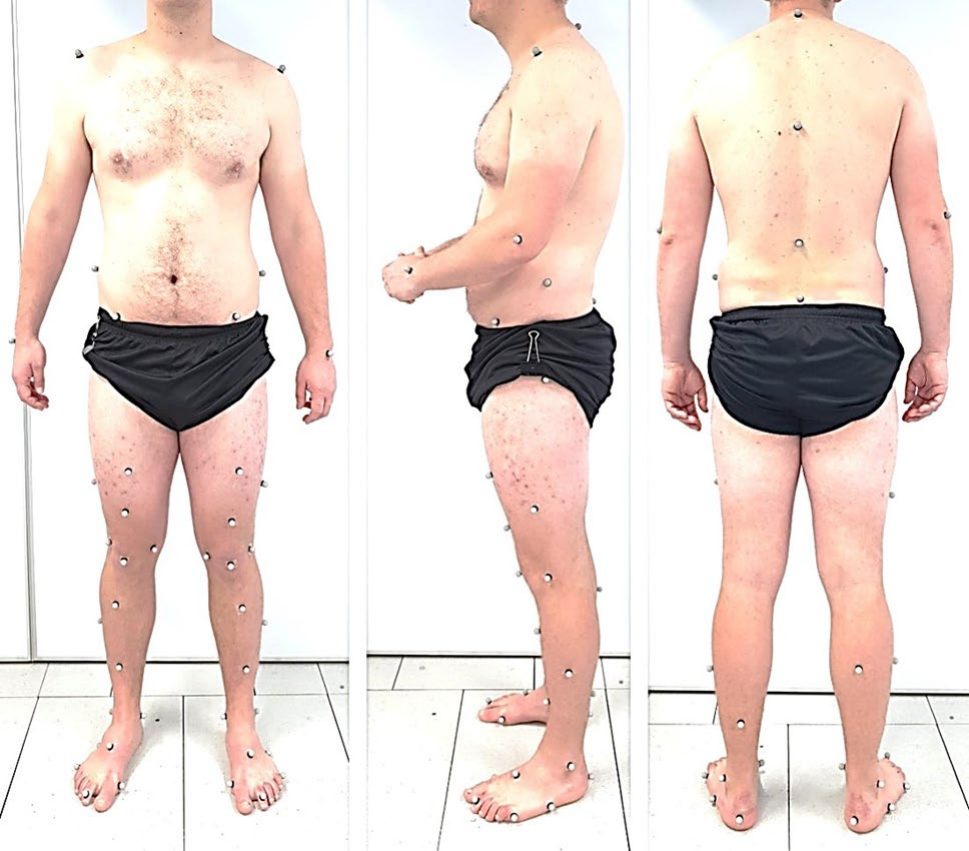
Marker placement using a modified Cleveland Clinic Markerset. Anatomical markers positioned on the right and left acromion, the right and left anterior superior iliac spines, the sacrum defined as the centre of the posterior superior iliac spines, upper border of the greater trochanters, medial and lateral epicondyles and malleoli and the 1st and 5th metatarsal heads. Additional markers ensured at least three tracking markers were available per segment. These were positioned on the heel, lateral and superior aspects of the foot, at C7, T9 and a cluster of three markers on the thighs and shanks

**Fig. 2:**
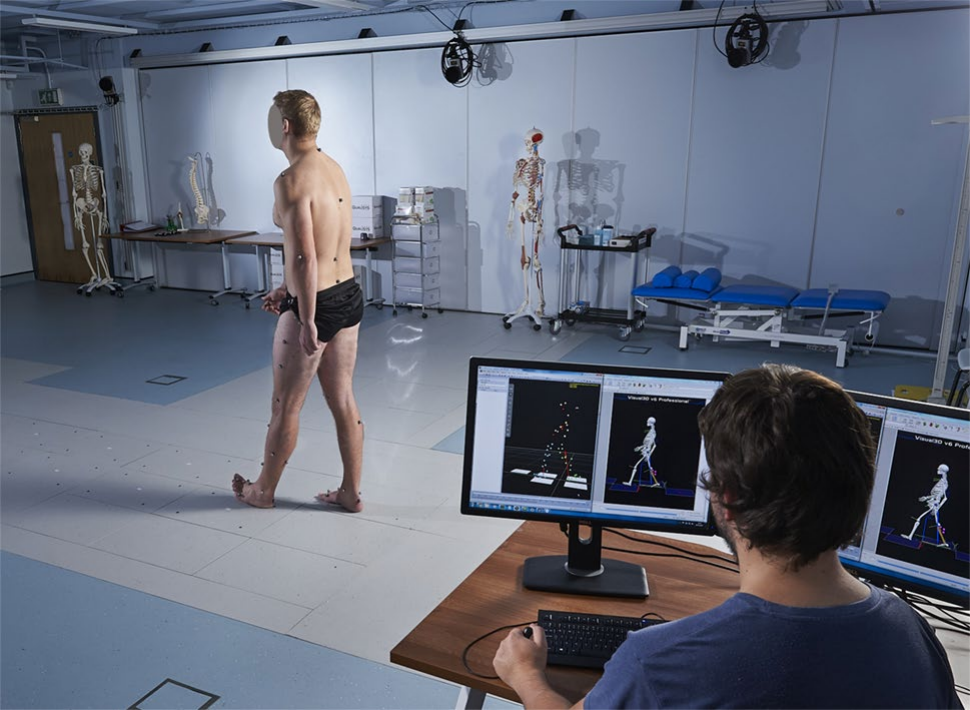
Data collection setup for gait analysis

Three-dimensional gait analysis was performed on patients before (average 1.7 ± 1.8 months) and after HTO surgery (average 13.8 ± 4.5 months) and at one time point for the control group. Patient-reported outcome measures (PROMs) including Oxford Knee Score (OKS) [21], Knee Outcome Survey (KOS) [13] and a visual analogue pain score were collected at each gait assessment (Table 2). Gait analysis was performed using an 8 Oqus camera system (Qualisys, Sweden) capturing at 120 Hz, synchronised with either two or four (due to laboratory upgrades) force platforms (Bertec Corp., USA) capturing at 1080 Hz. A modified Cleveland marker placement was applied, shown in Fig. 1, and subjects walked at their self-selected speed for a minimum of 6 successful trials. Where data issues or outliers were identified, a minimum of 3 trials were used in the analysis. Figure 2 shows the data collection setup.

### Biomechanical analysis

Joint kinematics, kinetics and temporal parameters were calculated within Visual 3D (C-Motion, USA) using a custom model of the lower limbs and thorax. A Butterworth fourth order filter was used on raw marker coordinate data with a cut-off frequency of 7 Hz (defined through a power spectral analysis of the marker data from all subjects and trials used in this analysis to ensure 99% accumulative power). Anatomical joint axes and their positional relationship to tracking markers used to define segment motion, were calculated from a measurement of static standing. Knee and ankle centres were defined as the midpoint of the epicondyles and malleoli, respectively. Hip joint centres were defined relative to the markers on the pelvis using the Bell regression model [3]. The thorax axis origin was defined between virtual iliac crest markers created from the position of the anterior superior iliac spine and trochanter markers. Local coordinate systems were defined to coincide with anatomic axes and segments defined as rigid bodies with inertial properties estimated according to [19].

Joint angles were calculated using the Cardan/Euler *x*, *y*, *z* sequence [11], equivalent to the Grood and Suntay definitions [18]. Angles are defined as the orientation of the distal segment with respect to the reference proximal segment, with the exception of the thorax and feet where they are calculated with respect to a virtual laboratory axes aligned with the direction of gait. Inverse dynamics was used to calculate net external knee joint moments represented in the tibial reference frame since loading at the knee joint is of primary interest [30]. All moments were normalised by the participant’s body weight times height to reduce differences due to gender [29].

**Fig. 3:**
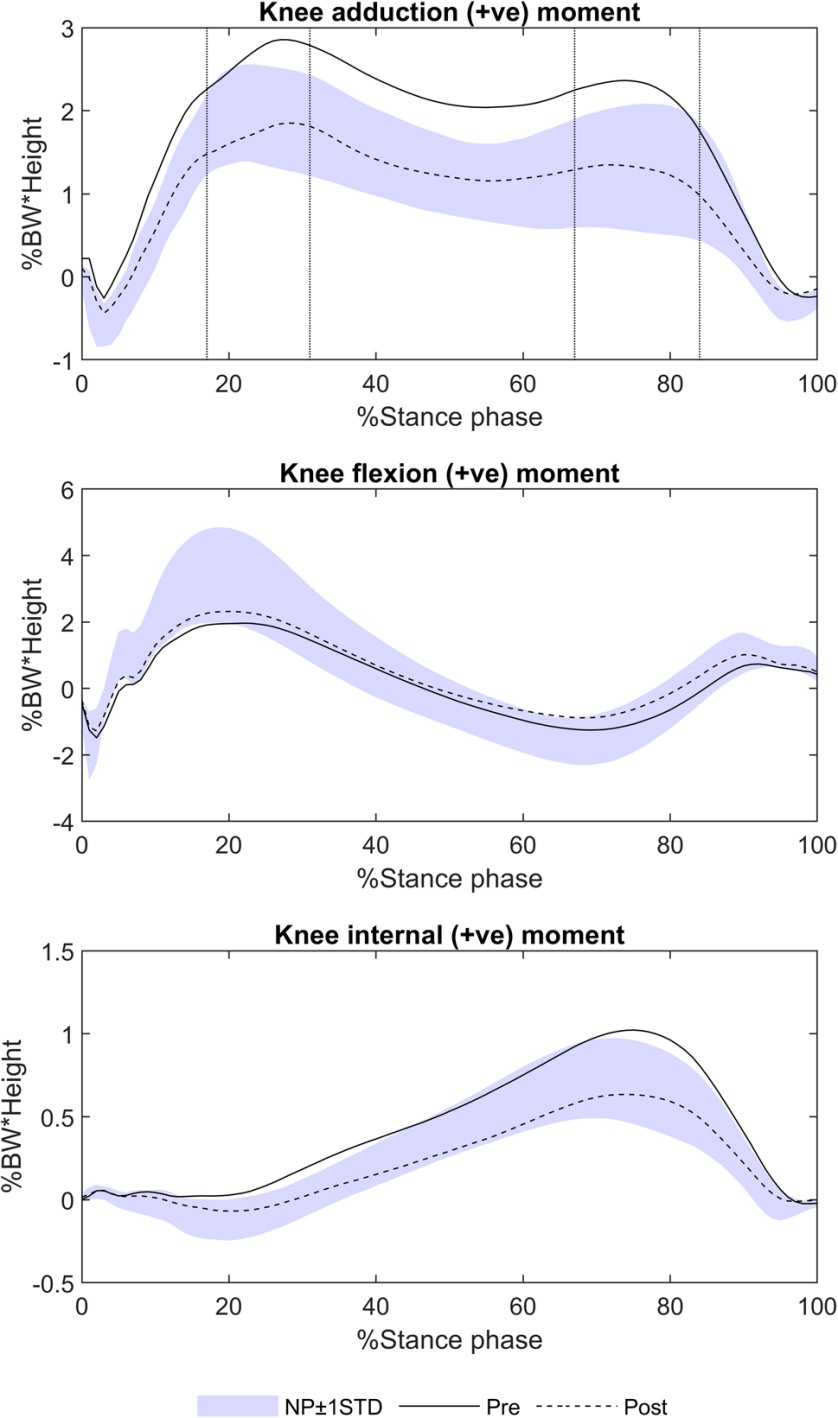
Moments acting at the knee during the stance phase of gait, and the location of EKAM P1 and P2. Top graph shows the knee adduction moment, indicating loading in the frontal plane. EKAM P1 was defined within 17% and 31% stance and EKAM P2 defined within 34% and 67%. These ranges were defined as the mean percentage in stance ± 2SD where the peaks occurred in 21 knees with bi-phasic patterns. Therefore, this approach provided a consistent way of defining maximum values when bi-phasic patterns did not exist. The middle graph shows the flexion/extension knee moment, indicating loading in the sagittal plane. The bottom graph shows the internal/external knee moment, indicating loading in the transverse plane

**Fig. 4:**
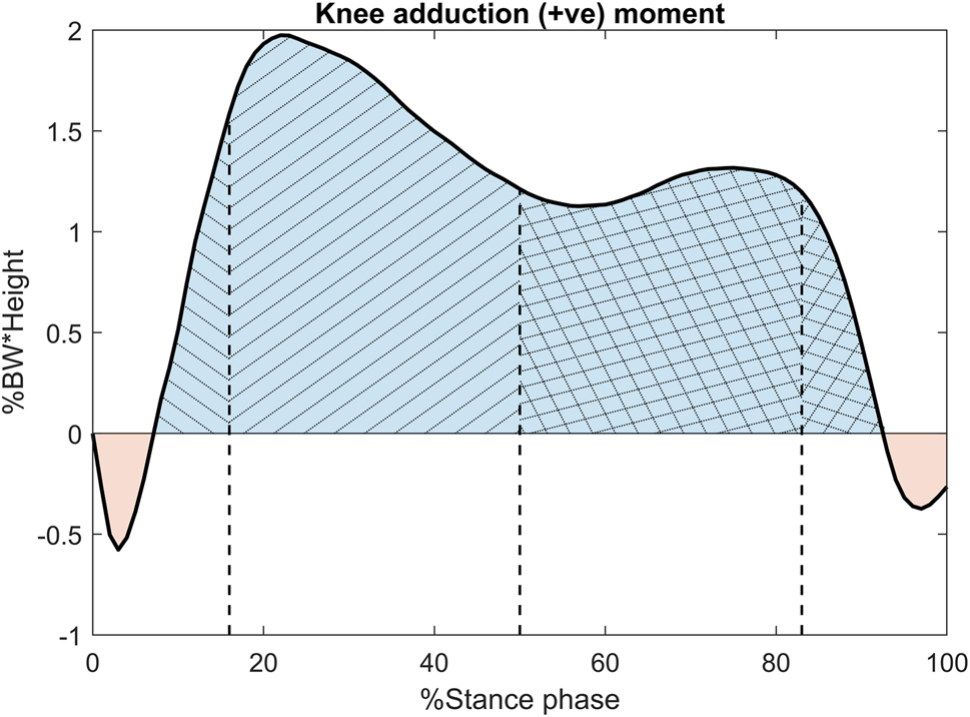
KAAI range definition. KAAI during stance phase is the area under the positive region of EKAM shaded in blue. This measure provides an indication of medial loading acting throughout the stance phase of gait. The first half of KAAI provides an indication of medial loading from heal strike to midstance. The second half of KAAI describes loading from midstance to toe off. The region from 0 to 16% describes loading response from initial contact and weight acceptance onto the supporting limb until the end of double limb support; 17%–midstance describes the action of the tibia rotating over the stationary foot until body weight is transferred to the forefoot; midstance–83% describes terminal stance where the heel rises to the beginning of terminal double limb support; 84–100% describes pre-swing where weight is being transferred onto the contralateral limb in preparation for swing. The red-shaded regions illustrate the abduction angular impulse during the first and second half of stance, indicating lateralised loading during initial contact and toe off

The two peaks of the external knee adduction moment were calculated for the first and second half of stance phase (EKAM P1, EKAM P2), along with the EKAM trough defined at 50% stance. Since not all EKAM moments were bi-phasic, EKAM P1 and EKAM P2 were defined as peaks within (17% and 31%) and (34% and 67%) of stance phase, respectively (Fig. 3). These ranges were defined as the mean percentage in stance ± 2SD where the peaks occurred in 21 knees with bi-phasic patterns. Knee angular impulses were calculated as the integral of the positive and negative regions of the moment profiles separately, in three clinical planes, to provide information on average loading over the stance phase. In the frontal plane, KAAI was also calculated during the first and second half of stance and for four additional portions of the stance phase [38] to identify when the largest effect on loading occurs following HTO surgery (Fig. 4). Joint knee powers were calculated in three clinical planes with positive and negative integrals determining positive and negative work done, respectively. Foot progression angle was calculated as the angle between the long axis of the foot and the line of forward progression. Varus angle ROM and frontal plane knee joint velocity during the loading phase from heel strike (HS) to 16% stance were computed to give an indication of frontal plane knee thrust [14]. Centre of pressure (COP) was calculated two ways at the average time of the EKAM peaks, defined from the subset of participants with bi-phasic EKAM patterns. COP calculated relative to the foot axis was normalised as a percentage of foot length (defined between the ankle and metatarsals) and foot width (defined between the 1st and 5th metatarsal). COP was also calculated as the distance in mm between the COP and knee centre measured in the laboratory reference frame aligned with the direction of gait.

### Statistical analysis

Paired samples t test was performed using SPSS version 23 (SPSS Inc., USA) to identify significant differences associated with HTO surgery. Where parametric assumptions were not met, a Wilcoxon signed-rank test was used. Independent *t* tests were used to determine significant differences in the pre- and post-HTO measurements compared to the control group. Where parametric assumptions were not met, a Mann–Whitney *U* test was performed. Significance was determined when *p* < 0.05 for all statistical tests. As EKAM and KAAI are considered the main parameters of interest, with others considered as part of exploratory analyses, a post hoc multiple testing correction was not performed. Sufficient power was confirmed using KAAI and EKAM data from [25].

## Results

Table 1 shows participant demographics and clinical measures. In Table 2, PROMs improved significantly as a result of HTO (*p* ≤ 0.002), however, they remained significantly different to the control subjects (*p* < 0.001).

**Table 1:** Demographic and clinical characteristics for patients at baseline and controls

Demographics	Controls Mean (SD)	Pre-HTO Mean (SD)	Pre-HTO vs controls *p* value
Number of knees	18	19	
Gender (M/F)	10/8	16/2	
Mean age, years (SD)	34.6 (11.2)	51.2 (7.0)	< 0.001**
Height, m (SD)	1.7 (0.1)	1.7 (0.1)	ns
Mass, kg (SD)	70.2 (12.9)	90.1 (22.5)	0.002**
BMI, kg/m^2^ (SD)	24.5 (4.1)	29.5 (5.8)	0.003^††^
KL Grade		2 KL2, 12 KL3, 5 KL4	
mTFA (°)		8.0 (3.6) post-HTO: 0.7 (2.9) for* n* = 16	

Significant difference (*p* < 0.01) indicated by ** where parametric or ^††^ where non-parametric tests used

*ns* no significance

**Table 2:** Patient-reported outcome measures

Scores	Controls Mean (SD)	Pre-HTO Mean (SD)	Post-HTO Mean (SD)	Controls vs pre-HTO *p* value	Controls vs post-HTO *p* value	Pre- vs post-HTO *p* value
Oxford Knee Score	47.7 (1.0) (*n* = 17)	26.6 (7.9) (*n* = 18)	36.8 (6.0) (*n* = 18)	< 0.001^††^	< 0.001^††^	< 0.001**
Knee Outcome Survey	79 .4 (1.2) (*n* = 16)	47.2 (11.8) (*n* = 18)	60.3 (9.9) (*n* = 18)	< 0.001^††^	< 0.001^††^	0.002**
Pain Score	0.4 (1.1) (*n* = 15)	49.4 (26.3) (*n* = 16)	15.4 (10.9) (*n* = 16)	< 0.001^††^	< 0.001^††^	< 0.001**

Significant difference (*p* < 0.01) indicated by ** where parametric or ^††^ where non-parametric tests used

Before surgery, compared to the control group, patients walked with significantly slower gait speed (*p* = 0.042). Differences in gait speed were nonsignificant after HTO (Table 3). Patients spent longer in the stance phase compared to the controls both pre- (*p* = 0.001) and post-HTO (*p* = 0.008).

**Table 3:** Temporal and kinematic parameters

Metrics	Control Mean (SD)	Pre-HTO Mean (SD)	Post-HTO Mean (SD)	Control vs pre-HTO *p* value	Control vs post-HTO *p* value	Pre- vs post-HTO *p* value
Gait speed (m/s)	1.2 (0.1)	1.0 (0.2)	1.1 (0.2)	0.042^†^	ns	0.014*
Stance percentage (%)	60.1 (1.5)	62.5 (2.5)	61.9 (2.3)	0.001**	0.008**	ns
Foot progression angle ( + ) = toe out (°)	13.1 (4.0)	14.6 (6.8)	15.3 (7.3)	ns	ns	ns
Stance width (m)	0.15 (0.03)	0.16 (0.03)^a^	0.17 (0.03)	ns	ns	ns
Trunk sway ROM (°)	3.3 (1.2)	5.0 (2.8)	4.1 (2.3)	0.031^†^	ns	0.007**
COP related to foot, 1st half stance						
Medial ( + ) lateral (−) (% foot width)	*−* 8.2 (3.3)	*−* 7.6 (4.3)	*−* 9.3 (3.5)	ns	ns	ns
Anterior ( + ) posterior (−) (% foot length)	22.4 (11.0)	27.2 (10.7)	24.6 (11.5)	ns	ns	ns
COP related to foot, 2nd half stance						
Medial ( + ) lateral (−) (% foot width)	1.7 (4.2)	− 1.0 (4.1)	− 1.1 (3.3)	ns	0.034*	ns
Anterior ( + ) posterior (−) (% foot length)	119.5 (8.6)	114.3 (7.7)	112.3 (8.2)	ns	0.014*	ns
COP related to the knee origin, 1st half stance, mm						
Medial ( + ) lateral (−)	− 0.7 (12.8)	18.7 (22.1)	− 1.1 (17.6)	0.003**	ns	< 0.001**
Anterior ( + ) posterior (−)	− 0.4 (21.7)	16.7 (23.5)	15.5 (19.6)	0.027*	0.025*	ns
COP related to the knee origin, 2nd half stance (mm)						
Medial ( + ) lateral (−)	− 8.0 (15.2)	8.1 (22.8)	− 12.7 (21.3)	0.017*	ns	< 0.001**
Anterior ( + ) posterior (−)	− 31.6 (12.9)	− 27.3 (22.5)	− 37.8 (19.3)	ns	ns	0.012*
Knee frontal plane ROM (°)	10.2 (2.8)	11.6 (3.8)	13.0 (3.5)	ns	0.013*	ns
Knee sagittal plane ROM (°)	64.6 (4.1)	57.9 (6.3)	59.5 (5.6)	< 0.001^††^	0.003**	0.021*
Knee transverse plane ROM (°)	16 .0 (4.2)	16.3 (4.0)	17.2 (3.9)	ns	ns	ns
Frontal plane dynamic knee alignment ROM, HS-16% (°)	2.4 (1.0)	3.2 (1.7)	3.2 (2.1)	ns	ns	ns
Peak knee adduction (+) velocity, HS-16% (°/s)	43.6 (29.1)	57.1 (35.3)	43.4 (19.3)	ns	ns	ns
Peak knee abduction (−) velocity, HS-16% (°/s)	− 32.5 (27.6)	− 29.0 (31.5)	− 40.6 (28.8)	ns	ns	ns

Significant difference (*p* < 0.05) and (*p* < 0.01) indicated by * and ** where parametric or ^†^ and ^††^ where non-parametric tests used

*ns *indicates no significance

^a^Data missing for one subject

### Kinematics

Compared to the control group, patients adopted a significantly larger frontal plane trunk sway ROM (*p* = 0.031) before, but not after HTO (ns). Sagittal plane knee ROM was smaller than controls pre- (*p* < 0.001) and post-HTO (*p* = 0.003) (Table 3). The presence of varus or valgus thrust was not confirmed.

### Knee loading

External knee moments are listed in Table 4 and displayed in Fig. 3. High EKAM measures were significantly reduced following HTO (*p* < 0.001) becoming nonsignificant compared to controls. Flexion moments remained consistently lower than controls both pre- (*p* = 0.005) and post-HTO (*p* = 0.016). An elevated internal rotation moment pre-HTO (*p* = 0.032) reduced significantly following HTO (*p* < 0.001).

**Table 4:** Knee moments

External knee moments, % BW.h	Controls Mean (SD)	Pre-HTO Mean (SD)	Post-HTO Mean (SD)	Control vs pre-HTO *p* value	Control vs post-HTO *p* value	Pre- vs post-HTO *p* value
Adduction (+) moment						
Maximum	2.18 (0.60)	3.15 (1.25)	2.11 (0.97)	0.005**	ns	< 0.001^††^
1st peak (1st half stance)	2.11 (0.60)	3.02 (1.25)	2.06 (0.96)	0.008**	ns	< 0.001^††^
2nd peak (2nd half stance)	1.44 (0.72)	2.46 (1.14)	1.49 (0.80)	0.003**	ns	< 0.001**
Midstance	1.21 (0.47)	2.07 (0.80)	1.23 (0.58)	< 0.001**	ns	< 0.001^††^
Flexion (+) moment peak	3.57 (1.41)	2.29 (1.16)	2.52 (1.05)	0.005**	0.016*	ns
Extension (−) moment peak	− 2.36 (0.79)	− 2.01 (0.76)	− 1.81 (0.78)	ns	0.042*	ns
Internal (+) rotation moment peak	0.76 (0.24)	1.05 (0.50)	0.68 (0.39)	0.032*	ns	< 0.001^††^
External (−) rotation moment peak	− 0.18 (0.10)	− 0.11 (0.09)	− 0.12 (0.09)	0.026^†^	ns	ns

Significant difference (*p* < 0.01) indicated by ** where parametric or ^††^ where non-parametric tests used

*ns* no significance

Table 5 displays knee angular impulses. KAAI during each portion of stance was statistically higher than controls pre-HTO (*p* ≤ 0.026) and restored to normal levels following HTO (*p* ≤ 0.002). A significant increase in the abduction angular impulse at the start of EKAM was also observed (*p* = 0.014). Post-HTO, the extension angular impulse reduced (*p* = 0.013) but remained within normal ranges and a high internal angular impulse (*p* = 0.002) reduced to normal levels (*p* < 0.001).

**Table 5:** Knee angular impulse

Knee angular impulse, %BW.h.s	Controls Mean (SD)	Pre-HTO Mean (SD)	Post-HTO Mean (SD)	Control vs pre-HTO *p* value	Control vs post-HTO *p* value	Pre- vs post-HTO *p* value
Adduction (+) angular impulse						
Stance	0.73 (0.28)	1.35 (0.54)	0.79 (0.39)	< 0.001**	ns	< 0.001**
1st half stance	0.42 (0.14)	0.73 (0.28)	0.45 (0.20)	< 0.001**	ns	< 0.001**
2nd half stance	0.31 (0.16)	0.61 (0.28)	0.35 (0.20)	< 0.001^††^	ns	< 0.001**
0–16% stance	0.05 (0.02)	0.11 (0.07)	0.06 (0.03)	0.002^††^	ns	< 0.001**
17%–midstance	0.36 (0.11)	0.61 (0.22)	0.38 (0.17)	< 0.001**	ns	< 0.001**
Midstance–83% stance	0.26 (0.13)	0.52 (0.23)	0.29 (0.16)	< 0.001^††^	ns	< 0.001**
84–100% stance	0.04 (0.02)	0.07 (0.05)	0.04 (0.03)	0.026*	ns	0.002**
Abduction (−) angular impulse in stance						
1st half stance	− 0.02 (0.01)	− 0.01 (0.01)	− 0.01 (0.01)	0.002^††^	ns	0.014^†^
2nd half stance	− 0.02 (0.01)	− 0.01 (0.01)	− 0.01 (0.01)	0.036^†^	ns	ns
Flexion (+) angular impulse	0.64 (0.26)	0.53 (0.40)	0.57 (0.30)	ns	ns	ns
Extension (−) angular impulse	− 0.30 (0.17)	− 0.37 (0.33)	− 0.25 (0.27)	ns	ns	0.013*
Internal (+) angular impulse	0.19 (0.07)	0.32 (0.15)	0.18 (0.11)	0.002**	ns	< 0.001**
External (−) angular impulse	− 0.02 (0.01)	− 0.01 (0.01)	− 0.02 (0.02)	0.049^†^	ns	ns

Significant difference (*p* < 0.05) and (*p* < 0.01) indicated by * and ** where parametric or ^†^ and ^††^ where non-parametric tests used

*ns* no significance

### Work done

More work was generated (*p* = 0.004) and absorbed (*p* = 0.012) in the frontal plane, and absorbed (*p* < 0.001) in the sagittal plane pre-HTO compared to controls (Table 6). These were restored to normal levels following HTO (*p* < 0.01). HTO surgery resulted in transverse plane energy generation (*p* = 0.008) and absorption (*p* < 0.001) reducing to levels below that of the control group.

**Table 6:** Knee powers

Work done at the knee, mJ/kg	Controls Mean (SD)	Pre-HTO Mean (SD)	Post-HTO Mean (SD)	Control vs pre-HTO *p* value	Control vs post-HTO *p* value	Pre- vs post-HTO *p* value
Frontal plane positive work	24.4 (16.4)	50.4 (43.1)	27.0 (16.5)	0.004^††^	ns	0.009^††^
Frontal plane negative work	− 20.1 (12.9)	− 32.6 (17.6)	− 18.9 (11.5)	0.012^†^	ns	< 0.001**
Sagittal plane positive work	83.4 (36.7)	68.7 (37.0)	57.3 (26.3)	ns	0.018*	ns
Sagittal plane negative work	− 137.5 (49.6)	− 84.0 (32.6)	− 106.7 (44.4)	< 0.001**	ns	0.007**
Transverse plane positive work	8.1 (4.0)	8.7 (5.2)	5.5 (3.6)	ns	0.007^††^	0.008**
Transverse plane negative work	− 11.8 (4.1)	− 14.7 (9.4)	− 8.0 (3.8)	ns	0.005**	< 0.001**

Significant difference (*p* < 0.05) and (*p* < 0.01) indicated by * and ** where parametric or ^†^ and ^††^ where non-parametric tests used

*ns* no significance

### COP measured relative to the foot

**Fig. 5:**
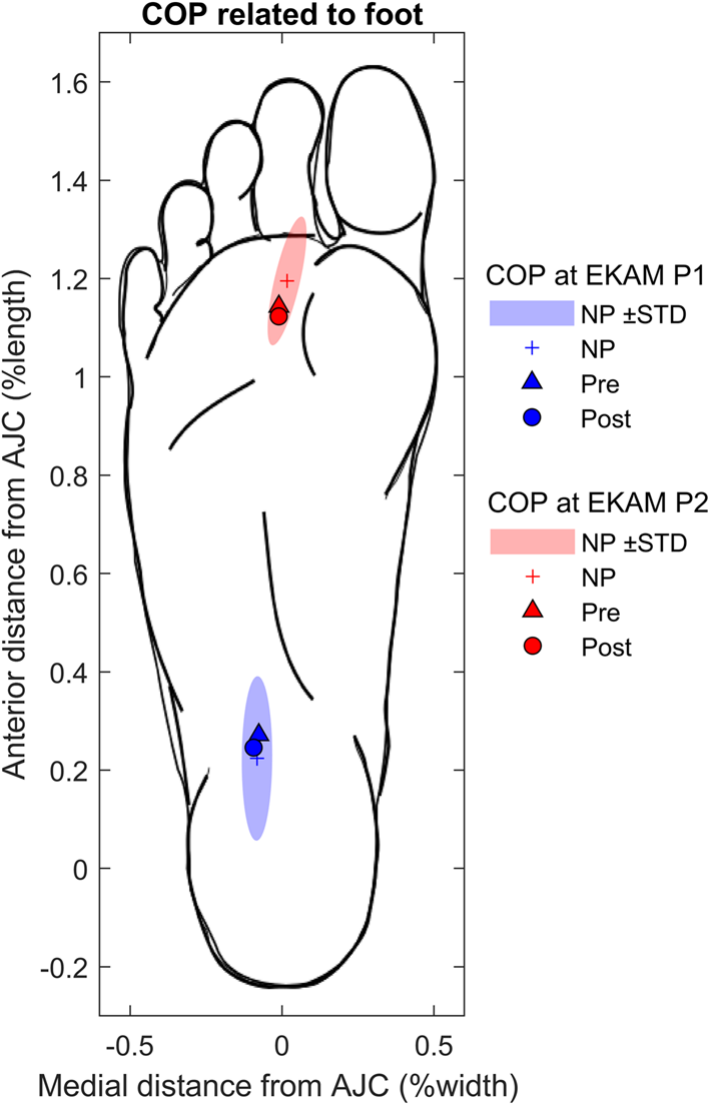
COP measured relative to the foot. COP measured pre- and post-HTO are illustrated at the timing of EKAM peak 1 in blue and EKAM peak 2 in red. The values are normalised to foot width (defined as the distance between the motion analysis markers positioned on the 1st and 5th metatarsal heads), and foot length (defined as the distance between the markers positioned on the metatarsal heads and malleoli). The COP is calculated in the foot coordinate system with the origin at the midpoint of the medial and lateral malleoli. An illustration of the foot is given to facilitate interpretation and is scaled to the average foot length and width for the subjects used in this study

Small differences in COP location relative to the control group were only significant post-HTO. However, there was no effect due to surgery as shown in Fig. 5 and Table 3.

### COP measured relative to the knee

**Fig. 6:**
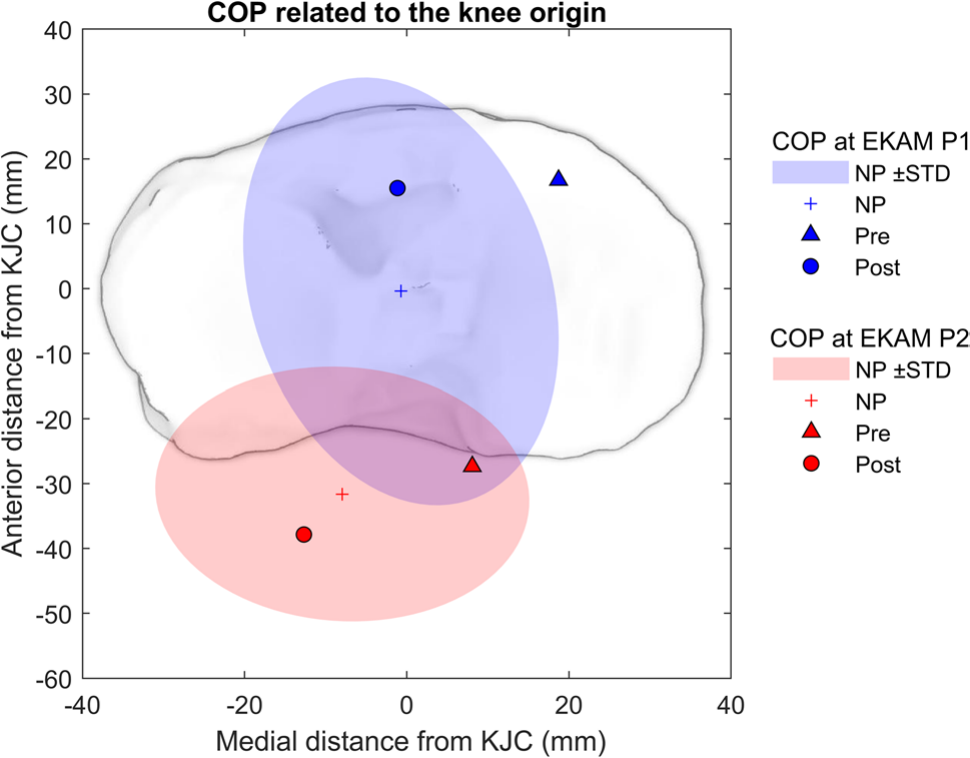
COP measured relative to the knee. COP measured pre- and post-HTO are illustrated at the timing of the EKAM peak 1 in blue and EKAM peak 2 in red. These were calculated in mm relative to the knee origin (defined as the midpoint of the medial and lateral epicondyles), in the laboratory reference frame aligned to the direction of gait. An illustration of the tibial plateau is given to facilitate interpretation and has been scaled to the average dimensions reported for adults within the study of [12]

HTO moved the COP laterally, which was observed at the timing of EKAM peak 1 and EKAM peak 2 (*p* < 0.001) (Fig. 6). At EKAM peak 1, the COP remained in a more anterior position to the knee centre both pre- (*p* = 0.027) and post-HTO (*p* = 0.025) compared to controls, due to a combination of the forefoot COP and a lower knee flexion angle. At EKAM peak 2, the COP location shifted posteriorly post-HTO (*p* = 0.012), but remained nonsignificant compared to the controls.

## Discussion

The most important finding of the present study was that HTO reduced frontal and transverse plane knee loading. This reduction is observed following surgery despite a significant increase in walking speed (*p* = 0.014), typically known to increase joint loading.

Overloading in the medial knee compartment pre-HTO, was reduced to normal levels by correcting the varus malalignment. In line with other studies [6, 26, 27], maximum EKAM, and the peaks in the first and second half of stance, and trough at 50% stance, were significantly reduced (*p* < 0.001) following HTO, by 33%, 32%, 39% and 41%, respectively.

The response of articular cartilage to mechanical load is not only affected by magnitude of load, but also the loading rate and duration [10]. For example, when high moments act over a longer duration, this may have important implications to cartilage degradation [4]. Pre-HTO, the KAAI calculated across the entirety of stance was significantly higher compared to levels found in the control group (*p* < 0.001); reducing by 41% (*p* < 0.001) to become ns compared to controls post-HTO. A reduction in KAAI as a result of HTO agrees with a previous study [6]. This reduction was consistent throughout the gait cycle. KAAI is known to be a better predictor of future OA progression than knee alignment alone, and it can be concluded that HTO is an effective method of reducing joint loading in-vivo, and thus provides effective mechanical protection for the degenerating medial compartment [4].

In the sagittal plane, the extension angular impulse decreased significantly after HTO (*p* = 0.013), though pre- and post-HTO values were nonsignificant compared to controls. In agreement with [26], varus malaligned patients with OA walk with reduced flexion moment peaks compared to controls (*p* = 0.005) and this is not corrected following HTO surgery (*p* = 0.016).

Early stance transverse plane loading was less than the control group pre-HTO, and this did not significantly increase post-HTO. In late stance, pre-HTO loading was higher than controls, indicated by higher peak internal rotation moment and internal angular impulse. Both decreased significantly (*p* < 0.001) becoming comparable to controls, in agreement with the study by [17].

The findings support the hypotheses that loading is altered in all three planes relative to controls pre-HTO and that HTO restores frontal plane loading to the level of control subjects, whilst also affecting load in the sagittal and transverse planes. Possible explanations for these findings are that improved alignment of the knee after surgery reduces out-of-plane knee loading during gait, hence improving the mechanical efficiency of gait. This is further supported by the normalisation of trunk lean (*p* = 0.007), previously identified as a gait modification strategy with high metabolic cost [9].

The finding of reduced knee joint loading following HTO corroborated with the knee joint power parameters. Work done at the knee was investigated to explore muscle performance in terms of energy generated and absorbed during joint motion in the stance phase. Positive (*p* = 0.004) and negative (*p* = 0.012) work done at the knee in the frontal plane was significantly higher pre-HTO compared to controls, as the pre-HTO subjects control the knee in greater varus alignment, returning to a normal level post-HTO (*p* < 0.01). In the transverse plane, pre-HTO energy absorption and generation were similar to control levels, decreasing significantly (*p* ≤ 0.008) as a result of surgery to a level significantly lower than controls (*p* ≤ 0.007). This may have important clinical implications. It is possible these differences reflect sub-optimal joint motion and/or muscle activity at the knee and normalisation of these features could further restore joint loading patterns and improve outcomes following HTO surgery.

Additional gait features known to influence EKAM magnitude were investigated, including foot progression angle, stance width and frontal plane trunk ROM. There was no significant difference in foot progression angle and stance width between patients and controls and this did not change significantly post-HTO. Preoperatively, patients used a significantly higher frontal plane trunk sway ROM (*p* = 0.031) than controls as a compensatory strategy to reduce medial compartment loading. This gait adaptation was not retained following HTO, in agreement with a previous study, and is representative of a more efficient gait pattern after surgery [6].

Motion of the knee during gait was altered in the sagittal plane for patients with knee OA and varus deformity, indicated by significantly smaller ROM compared to controls (*p* < 0.001). Although significant improvements were shown following HTO (*p* = 0.021), the hypothesis that all gait patterns would return to normal levels was disproven as sagittal plane ROM remained significantly lower than the control group (*p* = 0.003).

The presence of varus thrust represents a worsening of varus alignment during weight bearing. In contrast to the study by [14], the presence of varus thrust was not identified. A pairwise decrease in maximum, and an increase in minimum frontal plane velocity did approach significance, indicating there might be a shift from a varus towards a valgus thrust post-HTO. Due to large variations within the group, it is possible that some patients exhibited this gait feature as a load reducing compensatory mechanism, warranting further investigation. In addition, marker-based motion capture may not be a suitable measurement technique for detecting small changes in joint velocities due to errors recognised to be related to differentiation of marker displacement data.

The proposed biomechanical mechanism of HTO is to lateralise the COP relative to the knee joint centre (KJC) during gait, reducing frontal plane loading. The findings of this study confirm that the COP acts medial to the knee at the timing of both EKAM peaks pre-operatively and is shifted laterally (p < 0.001) with respect to the KJC following re-alignment surgery, thus reducing loading.

In the sagittal plane, the COP is positioned significantly more anterior to the knee at the first EKAM peak at weight acceptance for pre-HTO patients, and this feature is not corrected following surgery. This suggests the typical OA strategy of reducing knee extensor muscle demand and joint loading through reduced knee excursion during load acceptance [28] is still present post-HTO. This cohort appears to maintain a stiff-knee gait post-operatively which may result from weakness, instability, or retention of a pre-operative gait pattern.

Results from PROMs support the hypothesis, revealing clinically meaningful improvements to function and pain, despite scores not reaching the level of the control cohort. The reduction in pain is important to consider in more detail in future work as this may contribute to the improvement in mechanisms employed pre-HTO to reduce loading.

This work has demonstrated a number of clinically important findings. HTO is an effective operation at reducing both peak loading and knee angular impulse at the knee, in a way that would be expected to reduce structural disease progression in the future. Its effect is therefore not just pain relieving but is also likely to be protective against OA progression. After HTO, there are substantial biomechanical changes in all three planes which serve to make gait more efficient. However, gait does not fully return to normal after surgery, and there are persisting abnormalities which are important to understand as these may be addressed by targeted rehabilitation in the future.

A limitation to this work is the small cohort sizes which reflect the number of HTO surgeries performed in the UK. In addition, although providing comparisons with a control cohort is a study strength, participants are not matched for age, gender, or weight.

## Conclusions

In this cohort, HTO surgery successfully reduced both frontal and transverse plane joint loading. The location of COP relative to the knee was lateralised and patients reported significant improvements in pain and function. Reduced gait speed and increased trunk sway were naturally corrected post-HTO, however patients continued to spend a longer time in stance, walked with reduced sagittal range of motion and experienced reduced sagittal plane knee loading following surgery. Work done at the knee in the transverse plane reduced below normal levels post-HTO. Additional physiotherapy or gait retraining may provide a means to correcting remaining movement abnormalities and further work is required to address the biomechanical differences that remain for patients when compared to healthy controls, and their implications**.**

## References

[1] Andriacchi TP (1994). Dynamics of knee malalignment. Orthop Clin North Am.

[2] Baliunas AJ, Hurwitz DE, Ryals AB, Karrar A, Case JP, Block JA, Andriacchi TP (2002). Increased knee joint loads during walking are present in subjects with knee osteoarthritis. Osteoarthr Cartil.

[3] Bell AL, Brand RA, Pedersen DR (1989). Prediction of hip joint centre location from external landmarks. Hum Mov Sci.

[4] Bennell KL, Bowles K-A, Wang Y, Cicuttini F, Davies-Tuck M, Hinman RS (2011). Higher dynamic medial knee load predicts greater cartilage loss over 12 months in medial knee osteoarthritis. Ann Rheum Dis.

[5] Bhatnagar T, Jenkyn TR (2010). Internal kinetic changes in the knee due to high tibial osteotomy are well-correlated with change in external adduction moment: An osteoarthritic knee model. J Biomech.

[6] Birmingham TB, Giffin JR, Chesworth BM, Bryant DM, Litchfield RB, Willits K, Jenkyn TR, Fowler PJ (2009). Medial opening wedge high tibial osteotomy: A prospective cohort study of gait, radiographic, and patient-reported outcomes. Arthritis Rheum.

[7] Briem K, Ramsey DK, Newcomb W, Rudolph KS, Snyder-Mackler L (2007). Effects of the amount of valgus correction for medial compartment knee osteoarthritis on clinical outcome, knee kinetics and muscle co-contraction after opening wedge high tibial osteotomy. J Orthop Res.

[8] Brinkman J-M, Lobenhoffer P, Agneskirchner JD, Staubli AE, Wymenga AB, van Heerwaarden RJ (2008). Osteotomies around the knee: patient selection, stability of fixation and bone healing in high tibial osteotomies. J Bone Joint Surg Br.

[9] Caldwell LK, Laubach LL, Barrios JA (2013). Effect of specific gait modifications on medial knee loading, metabolic cost and perception of task difficulty. Clin Biomech Elsevier.

[10] Chen C-T, Burton-Wurster N, Lust G, Bank RA, Tekoppele JM (1999). Compositional and metabolic changes in damaged cartilage are peak-stress, stress-rate, and loading-duration dependent. J Orthop Res.

[11] Cole GK, Nigg BM, Ronsky JL, Yeadon MR (1993). Application of the joint coordinate system to three-dimensional joint attitude and movement representation: a standardization proposal. J Biomech Eng.

[12] Dai Y, Bischoff JE (2013). Comprehensive assessment of tibial plateau morphology in total knee arthroplasty: Influence of shape and size on anthropometric variability. J Orthop Res.

[13] Dawson J, Fitzpatrick R, Murray D, Carr A (1998). Questionnaire on the perceptions of patients about total knee replacement. J Bone Joint Surg Br.

[14] Deie M, Hoso T, Shimada N, Iwaki D, Nakamae A, Adachi N, Ochi M (2014). Differences between opening versus closing high tibial osteotomy on clinical outcomes and gait analysis. Knee.

[15] Elson DW, Petheram TG, Dawson MJ (2015). High reliability in digital planning of medial opening wedge high tibial osteotomy, using Miniaci’s method. Knee Surgery, Sport Traumatol Arthrosc.

[16] Golovakha ML, Orljanski W, Benedetto K-P, Panchenko S, Büchler P, Henle P, Aghayev E (2014). Comparison of theoretical fixation stability of three devices employed in medial opening wedge high tibial osteotomy: a finite element analysis. BMC Musculoskelet Disord.

[17] Goshima K, Sawaguchi T, Sakagoshi D, Shigemoto K, Hatsuchi Y, Akahane M (2017). Age does not affect the clinical and radiological outcomes after open-wedge high tibial osteotomy. Knee Surgery, Sport Traumatol Arthrosc.

[18] Grood ES, Suntay WJ (1983). A joint coordinate system for the clinical description of three-dimensional motions: application to the knee. J Biomech Eng.

[19] Hanavan EP (1964) A mathematical model of the human body. Aerospace Med. Res. Lab. Technical Report. Wright Patterson Air Force Base, Ohio 64:102

[20] Hernigou P, Medevielle D, Debeyre J, Goutallier D (1987). Proximal tibial osteotomy for osteoarthritis with varus deformity. A ten to thirteen-year follow-up study. J Bone Joint Surg Am.

[21] Irrgang JJ, Snyder-Mackler L, Wainner RS, Fu FH, Harner CD (1998). Development of a patient-reported measure of function of the knee. J Bone Joint Surg Am.

[22] Jackson JP, Waugh W (1961). Tibial osteotomy for osteoarthritis of the knee. J Bone Joint Surg Br Bone Joint J.

[23] Kellgren JH, Lawrence JS (1957). Radiological assessment of osteo-arthrosis. Ann Rheum Dis.

[24] Kutzner I, Trepczynski A, Heller MO, Bergmann G (2013). Knee adduction moment and medial contact force—facts about their correlation during gait. PLoS ONE.

[25] Lee SH, Lee O-S, Teo SH, Lee YS (2017). Change in gait after high tibial osteotomy: A systematic review and meta-analysis. Gait Posture.

[26] Lind M, McClelland J, Wittwer JE, Whitehead TS, Feller JA, Webster KE (2013). Gait analysis of walking before and after medial opening wedge high tibial osteotomy. Knee Surgery, Sport Traumatol Arthrosc.

[27] Marriott K, Birmingham TB, Kean C, Hui C, Jenkyn T, Giffin J (2015). Five-Year changes in gait biomechanics after concomitant high tibial osteotomy and ACL reconstruction in patients with medial knee osteoarthritis. Am J Sport Med.

[28] Mills K, Hunt MA, Ferber R (2013). Biomechanical deviations during level walking associated with knee osteoarthritis: a systematic review and meta-analysis. Arthritis Care Res.

[29] Moisio KC, Sumner DR, Shott S, Hurwitz DE (2003). Normalization of joint moments during gait: a comparison of two techniques. J Biomech.

[30] Mündermann A, Dyrby CO, Andriacchi TP (2005). Secondary gait changes in patients with medial compartment knee osteoarthritis: Increased load at the ankle, knee, and hip during walking. Arthritis Rheum.

[31] Noyes FR, Barber-Westin SD, Hewett TE (2000). High tibial osteotomy and ligament reconstruction for varus angulated anterior cruciate ligament-deficient knees. Am J Sports Med.

[32] Prodromos CC, Andriacchi TP, Galante JO (1985). A relationship between gait and clinical changes following high tibial osteotomy. J Bone Joint Surg Am.

[33] Ramsey DK, Snyder-Mackler L, Lewek M, Newcomb W, Rudolph KS, Statistical analysis Ramsey R (2007). Effect of anatomic realignment on muscle function during gait in patients with medial compartment knee osteoarthritis. Arthritis Rheum.

[34] Sharma L, Song J, Felson DT, Cahue S, Shamiyeh E, Dunlop DD (2001). The role of knee alignment in disease progression and functional decline in knee osteoarthritis. JAMA.

[35] Shelburne KB, Torry MR, Pandy MG (2006). Contributions of muscles, ligaments, and the ground-reaction force to tibiofemoral joint loading during normal gait. J Orthop Res.

[36] Teichtahl AJ, Cicuttini FM, Janakiramanan N, Davis SR, Wluka AE (2006). Static knee alignment and its association with radiographic knee osteoarthritis. Osteoarthr Cartil.

[37] Teichtahl AJ, Davies-Tuck ML, Wluka AE, Jones G, Cicuttini FM (2009). Change in knee angle influences the rate of medial tibial cartilage volume loss in knee osteoarthritis. Osteoarthr Cartil.

[38] Thorp LE, Sumner DR, Block JA, Moisio KC, Shott S, Wimmer MA (2006). Knee joint loading differs in individuals with mild compared with moderate medial knee osteoarthritis. Arthritis Rheum.

[39] Wang JW, Kuo KN, Andriacchi TP, Galante JO (1990). The influence of walking mechanics and time on the results of proximal tibial osteotomy. J Bone Joint Surg Am.

[40] Zhao D, Banks SA, Mitchell KH, D’Lima DD, Colwell CW, Fregly BJ (2007). Correlation between the knee adduction torque and medial contact force for a variety of gait patterns. J Orthop Res.

